# Coexistence of cryoglobulinemia and ANCA-associated vasculitis in a chronic brucellosis patient -a case report and literature review

**DOI:** 10.1186/s12879-023-08232-w

**Published:** 2023-05-02

**Authors:** Xu Yang, Congcong Jiao, Xiaomei Liu, Yongzhe Zhang, Hua Zhou, Yanqiu Wang

**Affiliations:** Department of Nephrology, Shengjing Hospital of China Medical University, 36 Sanhao Street, Shenyang, 110004 China

**Keywords:** Brucellosis, Glomerulonephritis, Renal insufficiency, ANCA, Cryoglobulinemia

## Abstract

**Background:**

The renal involvement of brucellosis is not common. Here we reported a rare case of chronic brucellosis accompanied by nephritic syndrome, acute kidney injury, the coexistence of cryoglobulinemia and antineutrophil cytoplasmic autoantibodies (ANCA) associated vasculitis (AAV) superimposed on iliac aortic stent implantation. The diagnosis and treatment of the case are instructive.

**Case presentation:**

A 49-year-old man with hypertension and iliac aortic stent implantation was admitted for unexplained renal failure with signs of nephritic syndrome, congestive heart failure, moderate anemia and livedoid change in the left sole with pain. His past history included chronic brucellosis and he just underwent the recurrence and completed the 6 weeks of antibiotics treatment. He demonstrated positive cytoplasmic/proteinase 3 ANCA, mixed type cryoglobulinemia and decreased C3. The kidney biopsy revealed endocapillary proliferative glomerulonephritis with a small amount of crescent formation. Immunofluorescence staining revealed only C3-positive staining. In accordance with clinical and laboratory findings, post-infective acute glomerulonephritis superimposed with AAV was diagnosed. The patient was treated with corticosteroids and antibiotics and sustained alleviation of renal function and brucellosis was achieved during the course of a 3-month follow-up.

**Conclusions:**

Here we describe the diagnostic and treatment challenge in a patient with chronic brucellosis related glomerulonephritis accompanied by the coexistence of AAV and cryoglobulinemia. Renal biopsy confirmed the diagnosis of postinfectious acute glomerulonephritis overlapping with ANCA related crescentic glomerulonephritis, which was not ever reported in the literature. The patient showed a good response to steroid treatment which indicated the immunity-induced kidney injury. Meanwhile, it is essential to recognize and actively treat the coexisting brucellosis even when there are no clinical signs of the active stage of infection. This is the critical point for a salutary patient outcome for brucellosis associated renal complications.

**Supplementary Information:**

The online version contains supplementary material available at 10.1186/s12879-023-08232-w.

## Background

Brucellosis is a zoonotic gram-negative bacterial infection which could affect multiple systems. It is still thought to be one of the most prevalent infectious diseases worldwide, especially in developing countries [1]. It could be divided into acute, subacute and chronic infection according to the duration of the disease [2]. The diverse manifestations of brucellosis made it difficult to diagnose especially in the non-epidemic areas.

The urinary system is not commonly affected in brucellosis. Only a very tiny amount of cases with prominent renal injury associated with brucellosis were consulted with the nephrologist and had been reported anecdotally [3–5]. Antineutrophil cytoplasmic autoantibodies (ANCA) are a group of autoimmune antibodies that are specific for the diagnosis of ANCA-associated vasculitis (AAV), which is an autoimmune disorder affecting small-sized, to a lesser degree, medium-sized vessels and renal injury is common in AAV which characterized by crescent formation with pauci-immune deposits [6, 7]. ANCA are also found in the occasions of tumors [8], infections [9], other autoimmune diseases, such as systemic lupus erythematosus [10], or induction by certain medicine [11]. But AAV caused by brucellosis was rarely reported. Cryoglobulins are a group of immunoglobulins that are characterized by precipitating at a temperature less than 37℃ and resolving in body temperature. They are classified into 3 types according to the composition of the immunoglobulins [12]. Mixed cryoglobulinemia has been associated with chronic infections of the Hepatitis C virus (HCV) and other pathogens [13, 14], but few were reported with brucellosis. The renal involvement in chronic brucellosis, accompanied by cryoglobulinemia coexistence with AAV is scarce, not to mention that happened in a patient with iliac aortic stent implantation.

Here we presented a rare case of chronic brucellosis with acute nephritic syndrome accompanied by cryoglobulinemia, AAV, superimposed on iliac aortic stent implantation. The diagnosis and treatment of this case were intricate and experiences in such a case are valuable.

## Case presentation

A 49-year-old male was admitted to Nephrology Department for oedema for 1 month and dyspnea for 1 week, with painful livedo reticularis in his left foot. His past history included recurrent episodes of brucellosis over the past four years. Two months prior to admission, brucellosis reoccurred and doxycycline and rifampin had been taken for 6 weeks and his symptoms of fever, sweating and general malaise disappeared. He had hypertension for 10 years, treated with telmisartan, nifedipine and indapamide and his blood pressure was about 150/90 mmHg. He underwent an iliac aneurysm stent implantation 6 years ago. He self-reported no evidence of renal disease before.

**Fig. 1:**
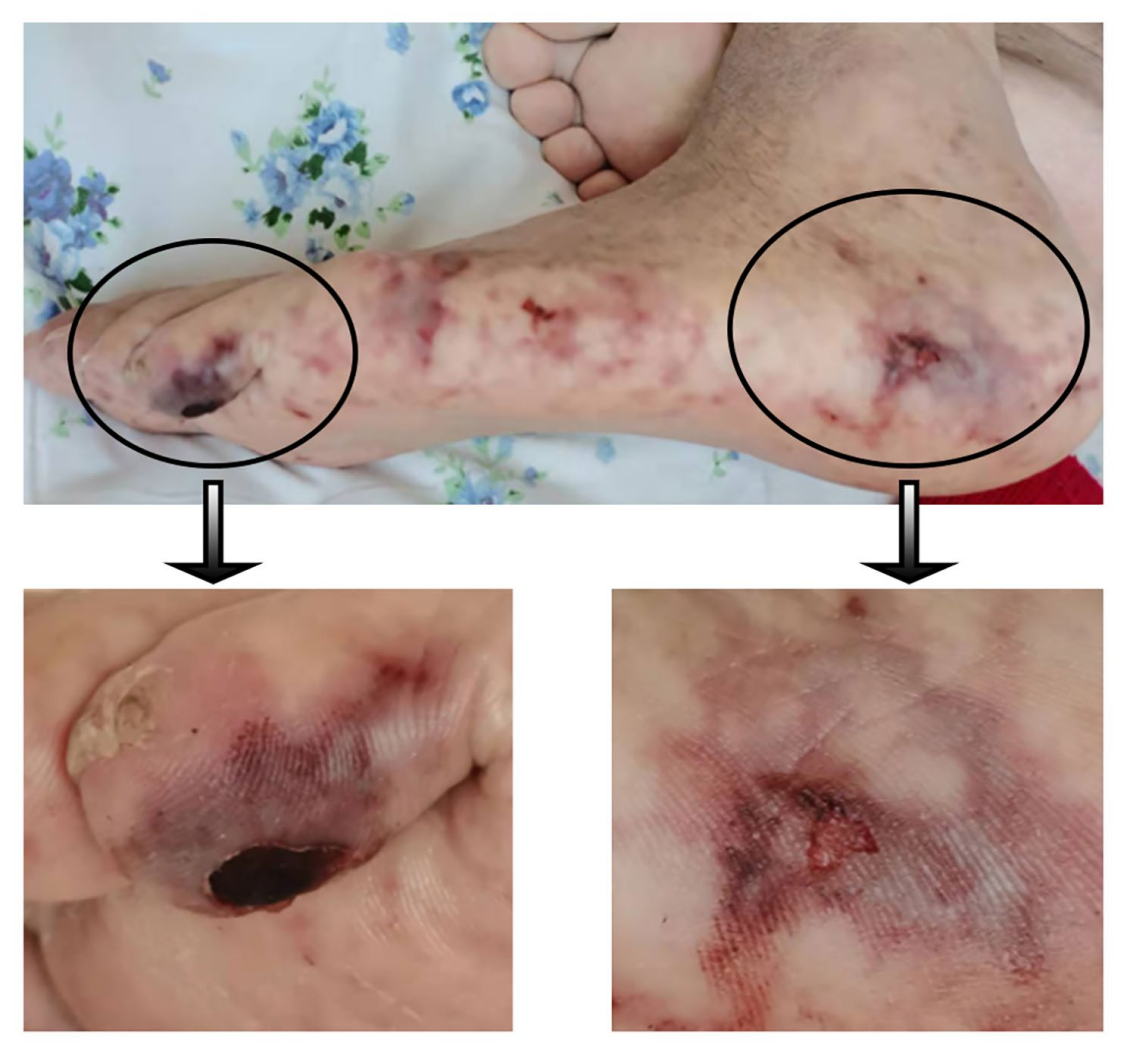
The livedoid changes in the left sole

On admission, the patient had no fever, and his blood pressure was 163/111 mmHg. Clinical examination revealed a puffy and pale appearance, general oedema, weakened breath sound in the bottom of both lungs and moist rales heard in both lungs. The ischemic manifestation occurred in the left lower limb including cold skin temperature and pulseless in arteria dorsalis pedis. Livedoid changes in the left sole were in Fig. 1.

**Fig. 2:**
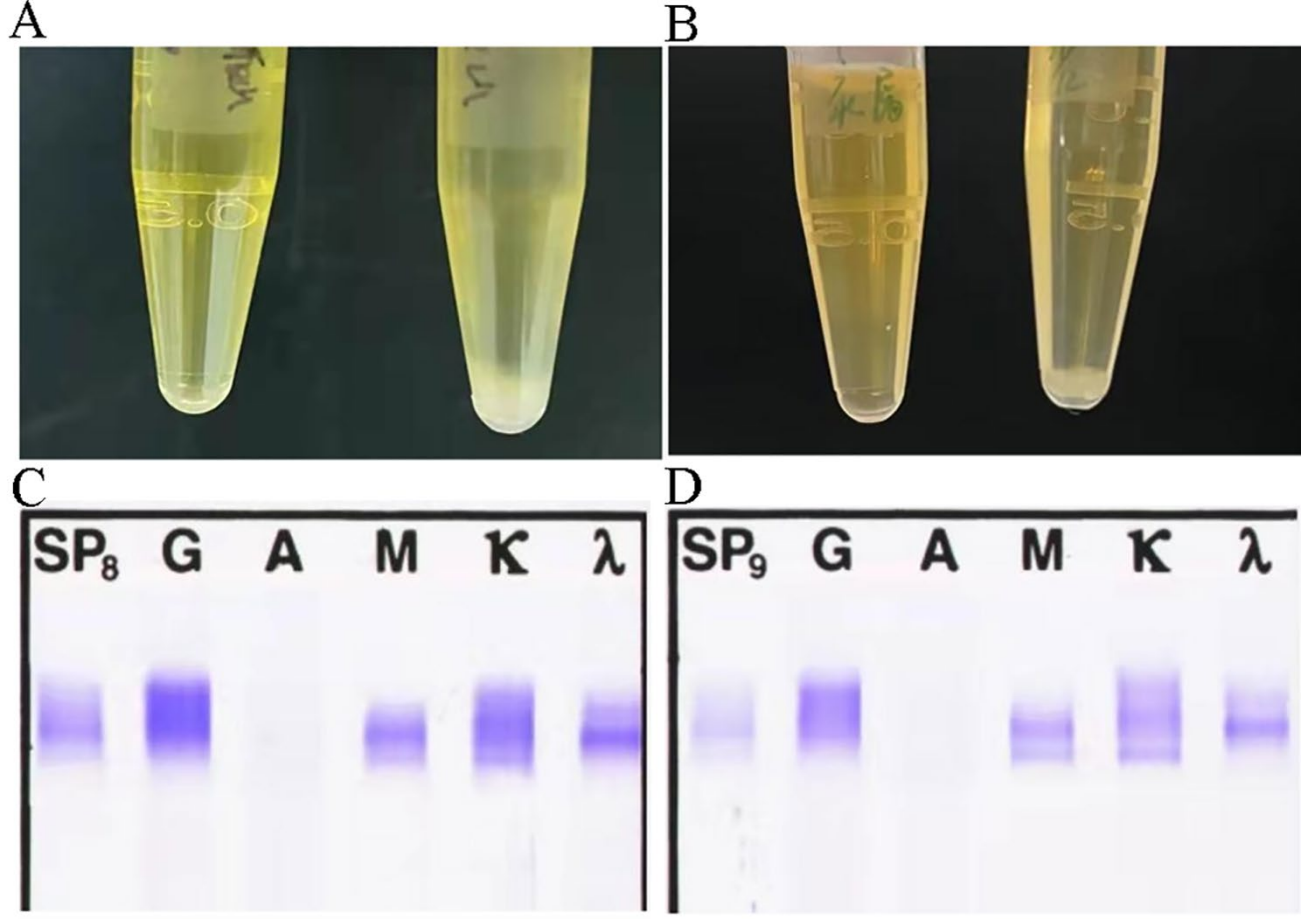
A: The plasma of health control (left) and patient with cryoglobulinemia (right) before treatment was exhibited. B: The plasma of health control (left) and patient with cryoglobulinemia (right) after treatment was exhibited. C: The immuno-electrophoresis of cryoglobulin before treatment was exhibited. D: The immuno-electrophoresis of cryoglobulin after treatment was exhibited. The original data of C and D were provided in supplemental material (Fig. 1S).

Laboratory tests showed elevated blood white blood cell (WBC) count with increased neutrophil ratio (WBC: 13.2 × 109/L and 90% neutrophil). The hemoglobin was 8.1 mg/dL. Urinalysis showed 2 + protein with dysmorphic red blood cells (77–430 /HP). Total urine protein excretion was 2.1 g/day. Blood urea nitrogen was 20.3 mmol/L, serum creatinine 203 µmol/L and creatinine clearance 31.9 mL/min/1.73 m^2^. Serum albumin was 23.4 g/L. Serum brain natriuretic peptide was 4087.3 µg/mL. Parathyroid hormone was 219.3 pg/mL. C-reaction protein was 53.2 g/L. Hypocomplementemia with C3 0.197 g/L and C4 0.101 g/L. Rheumatoid factor, anti-nuclear antibody IgG, cytoplasmic ANCA and proteinase 3 ANCA were positive. Reversible cryoprecipitate appeared and serum cryoglobulin level was 1.53 g/L. Immunoelectrophoretic analysis of the serum cryoglobulin showed the mixed polyclonal IgG and IgM (Fig. 2). Brucella serum agglutinins test (SAT) was positive at a titer of 1:200 and both the blood and the bone marrow culture were negative.

The pulmonary computed tomography (CT) scan showed a bilateral exudative lesion, bilateral pleural effusion and pulmonary atelectasis with pericardial effusion. The abdominal CT showed peritoneal and pelvic effusion, diffused abdominal wall oedema, metal stents image in the abdominal aorta, bilateral common iliac arteries and left external iliac artery. The electrocardiogram showed sinus rhythm with left ventricular high voltage. Ultrasonographic imaging of the kidneys revealed normal-sized kidneys with increased parenchymal echogenicity. The transthoracic echocardiogram detected enlargement in all four chambers and myocardial wall hypokinesia with decreased ejection fracture of 41%. Other changes included moderate pulmonary artery hypertension and a small amount of pericardial effusion with no vegetation on the cardiac valves. Pathological examination of the bone marrow showed hyperplasia of bone marrow with a normal ratio of granulocytes to erythrocytes. No poisoning particle was present in granulocytes. no parasites or bacteria were found.

**Fig. 3:**
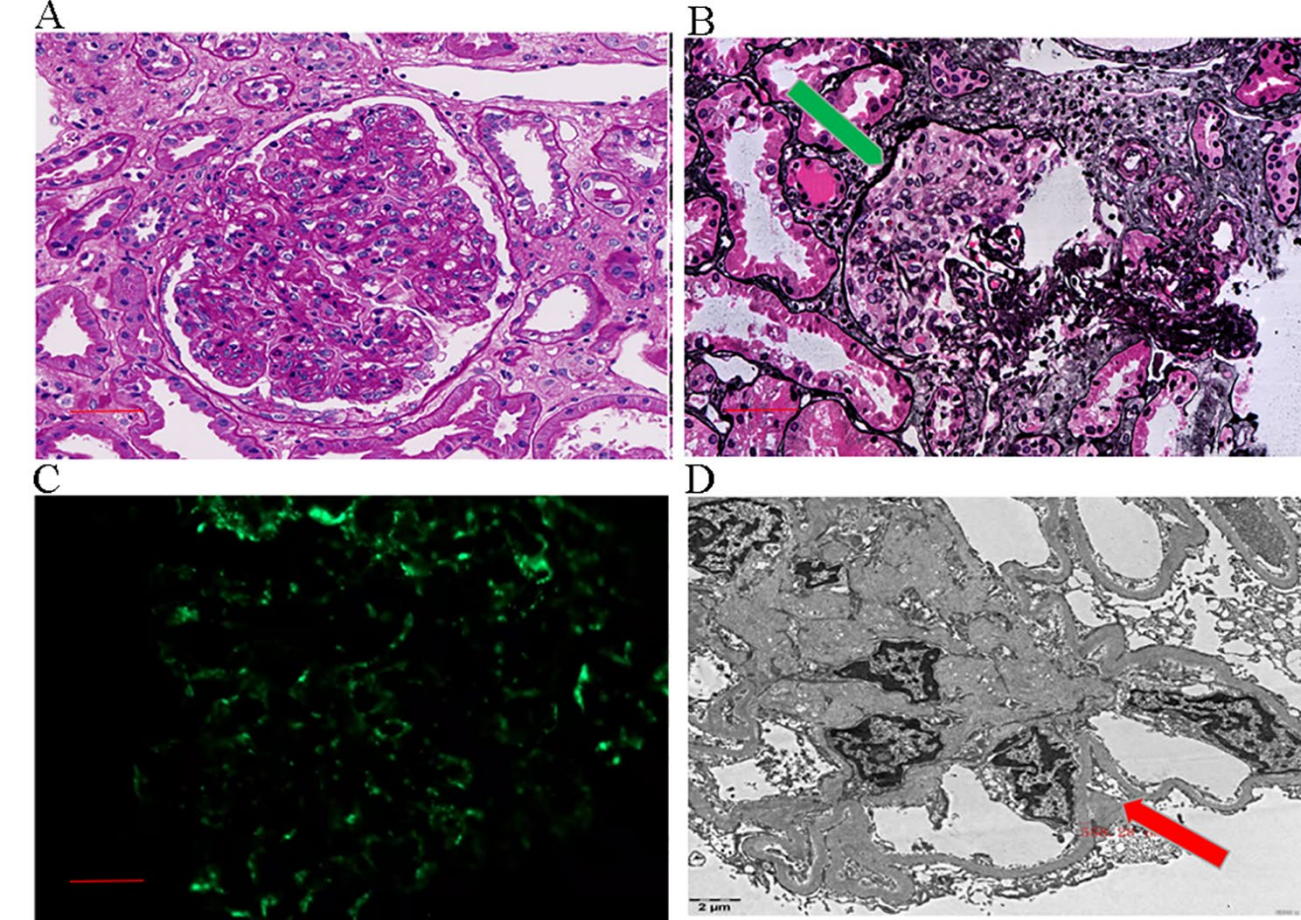
A: The diffused mesangial cells and matrix proliferation in the glomeruli were found under the light micrograph (PAS stain, 400×). B: The cellular crescent formation (green arrow) with crashed glomeruli under the light micrograph (PASM stain, 400×). C: Immunofluorescence exhibited strong granular C3 staining along the glomerular capillary. (400×). D: The hump-like electron-dense deposit (red arrow) under epithelial cells was observed by electron micrograph (6000×)

The patient received continuous renal replacement therapy (CRRT) to relieve the dyspnea and heavy oedema. A renal biopsy was performed after the patient’s condition become stable. The renal pathology revealed endocapillary proliferative glomerulonephritis with crescent formation. Hypertensive renal injury was also prominent (Fig. 3). On light microscopy, 32 glomeruli were identified. Glomerular sclerosis in 11 glomeruli. Hypercellularity in the remaining glomeruli, mainly endothelial cells and mesangial cells with a few neutrophils. A cellular, a fibrinous and a small cellulofibrous crescent were found. No basement membrane thickening. Severe vacuole and granular denaturation in tubular epithelial cells. 40% tubular atrophy, mild tubular interstitial oedema and multiple inflammatory cells infiltration with fibrosis. Thickening and narrowing of arteriole wall with segmental hyalinization. The immunofluorescence revealed diffuse strong C3 deposits along the capillary wall. No other immunoglobulin or complement deposit was present. Hump-like electron-dense deposits were found under epithelial cells by electron microscopy superimposed on hypertensive renal injury contributed to the patient’s renal involvement.

The patient was diagnosed with acute post-infectious glomerulonephritis (APIGN), AAV- related glomerulonephritis, secondary cryoglobulinemia, acute kidney injury on chronic kidney disease, primary hypertension Grade 3 (very high risk), congestive heart failure. Methylprednisolone (40 mg/QD/IV) was given on the 3rd day of admission to treat the acute nephritic syndrome. More important was the presence of ANCA and other immunological disorders and the severe foot pain and livedo reticularis which was a skin vasculitis related to cryoglobulinemia. And all the symptoms of the patient were relieved promptly and no need for CRRT treatment. Oral prednisone 50 mg per day was used and the patient was discharged from the hospital. For the treatment of brucellosis, anti-brucellosis treatment was suggested at the same time though the patient had no sign of active infection. Doxycycline and rifampin were added with a prolonged course of 6 months.

The patient recovered soon and on the 1st follow-up one month later, oral cortisone began to taper. 2 months later the prednisone was tapered to 30 mg/day and on the 3rd month, prednisone was 10 mg per day. The patient felt well and had no oedema, no fever and no dyspnea. The foot pain and livedo reticularis disappeared. There were little proteinuria and a slightly elevated creatinine level left. Now the patient is still under intensive follow-up. The biochemical examination records were listed in Table 1.

**Table 1:** The biochemical examination records

	On admission	On discharge	1 Month	3 Months	Normal range
Blood					
TP	55.1	57.9	63	67.3	60–83 g/L
Alb	23.4	30.8	39.4	41.7	35–53 g/L
Cre	287.9	101.2	105.8	109.5	59–104 umol/L
BUN	20.4	12.6	15.36	10.78	3-9.2 mmol/L
UA	591	426	454	422	208–428 umol/L
ANA	1:160	ND	ND	Negative	Negative
c-ANCA	Positive	Positive	Positive	Positive	Negative
PR3-ANCA	170.1	110.5	63.5	44.1	< 20 CU
CRP	53.2	9.67	4.79	1.86	0–8 mg/L
C3	0.197	0.433	0.606	0.649	0.74–1.4 g/L
C4	0.101	0.116	0.165	0.209	0.12–0.36 g/L
RF	2370	1620	839	368	0–30 IU/ml
IgG	18.4	12.5	12.5	11.5	6.95-15.15 g/L
IgM	4.16	3.73	3.23	1.94	0.4–1.59 g/L
Cryoglobulin	1.53	0.72	ND	ND	- g/L
BNP	4450.4	12,961	ND	148.9	0–80 pg/mL
WBC	8.28	7.58	5.86	3.81	3.5–9.5 10^9/L
Hb	67	69	98	100	130–172 g/L
PLT	106	153	153	128	135–350 10^9/L
Urine					
URTP	2.14	2.8	0.26	0.47	0.00-0.15 g/24 h
Pro	2+	2+	+/-	+/-	Negative
RBC	77.8	123.2	28.4	15.25	0.1–2.2 /HP
WBC	3	4.3	1.6	0.74	0.1–2.2 /HP

Alb albumin; ANA anti-nucleic antibody; BNP B-type natriuretic peptide; BUN blood urea nitrogen; C3 complement 3; C4 complement 4; c-ANCA cytoplasmic ANCA; Cre creatinine; CRP C-reaction protein; Hb hemoglobin; IgG immunoglobulin G; IgM immunoglobulin M; PLT platelet; PR3-ANCA proteinase 3 ANCA; Pro protein; RBC red blood cell. RF Rheumatoid factor; TP total protein; UA uric acid; URTP urinary total protein; WBC white blood cell

Antihypertensive treatment was given at the very beginning. Sacubitril/valsartan was given to reduce the elevated blood pressure and reverse the enlarged ventricle accompanied by amlodipine and arotinolol. Now the sacubitril/valsartan was used at the maximal dose (200 mg bid) and blood pressure is well controlled under 130/80 mmHg. The Echocardiography re-checked 3 weeks later showed left ventricular enlargement only and improved ventricular motion with an improved ejection fraction of 53%. There was still 5–8 mm pericardial effusion and no pulmonary hypertension.

The patient worked as a shepherd from 2012 to 2015. But he was first diagnosed with brucellosis 2 years after ceasing sheep husbandry with classical symptoms of fever, sweating, muscle pain and ankle pain. After combined use of oral doxycycline and rifampin for 6 weeks. His symptoms disappeared completely. But his symptoms were repeated another 3 times. Each time he was treated with the same therapeutic regimen and responded well. But his brucella antibody persists positive which may be a sign of inadequate treatment.

## Discussion and conclusions

Here we reported a rare case of chronic brucellosis associated with nephritic syndrome with renal insufficiency accompanied by cryoglobulinemia coexisting with positive ANCA, who underwent iliac angioplasty and stents implantation before. The coexistence of ANCA and cryoglobulinemia indicated immunological disturbance induced by the recurrent attack of brucellosis. Livedoid change in volar with pain, hypocomplementemia and positive RF are usually seen in cryoglobulinemia-related vasculitis, but not in AAV [15–17]. Renal pathology revealed endocapillary proliferative glomerulonephritis with the crescent formation which was diagnosed as a coexistence of APIGN and ANCA related glomerulonephritis. But there was no typical pathologic change of cryoglobulinemia-related renal injury. Corticosteroids was administrated accompanied by a prolonged course of anti-brucellosis treatment and the renal function recovered soon with proteinuria decreased significantly as well as no sign of relapse of Brucella infection. The process of diagnosis and treatment of the patient was roundabout and instructive.

Glomerulonephritis associated with brucellosis rarely happens. The renal pathologic change in Brucella infection varied from case to case. According to the previous report, the renal involvement of brucellosis could be categorized into 4 types: acute interstitial glomerulonephritis, chronic granulomatous interstitial nephritis, similar to renal tuberculosis, the renal abscess, and the glomerulonephritis with or without endocarditis [3]. For the last type, membranoproliferative nephritis, IgA nephritis and massive proliferative glomerulonephritis were all reported [18–20]. Rare pathologic changes include membranous nephropathy and minimal change disease [21, 22]. In our case, renal pathology revealed endocapillary proliferative glomerulonephritis with crescent formation. C3 deposit in the immunofluorescence and hump-like electron-dense deposits under epithelial cells in the electron microscope strongly indicated APIGN. The crescent may cause by ANCA-related glomerulonephritis which is with oligo-immune complex deposit. But there are no signs of cryoglobulin-related renal injury.

It should be distinguished from C3 glomerulopathy, which is a group of rare complement dysregulation renal diseases characterized by membranoproliferative glomerulonephritis (MPGN) with C3 deposit only. The two major subgroups of C3 glomerulopathy are dense deposit disease and C3 glomerulonephritis, which compose the type II and type III MPGN. But unlike type I MPGN, proliferation and insertion of mesangial cells in C3 glomerulonephritis is not so obvious and neutrophil infiltration and crescent formation can also present, which makes it difficult to be separated with APIGN with only C3 deposit that can be seen in the late stage of infection [23]. In the clinic, both of them share the manifestation of nephritic/nephrotic syndrome, hypocomplementemia, hypertension and renal insufficiency. However, hump-like deposits under epithelial cells in an electron microscope strongly support the diagnosis of APIGN [24]. Occupational exposure to aborted sheep and a history of chronic brucellosis is the backer of APIGN and the good response to steroid treatment also favors the diagnosis of APIGN. Usually, C3 glomerulopathy is refractory nephropathy unresponsive to immunosuppressants and chronic renal insufficiency is unavoidable. The presence of C3Nef and H factor may indicate C3 glomerulonephritis [25, 26].

Acute glomerulonephritis occurs in chronic brucellosis infection is rare. In our case, the patient suffers from chronic brucellosis and general oedema and oliguria developed in the patient after completion of antibiotic treatment of brucellosis for the 4th time and the patient apparently “healed”, which makes it difficult to connect these two events together. Simella Provatopoulou et al. reported a similar case of chronic brucellosis who developed nephrotic syndrome with mild renal insufficiency 2 months after his last episode of brucellosis. Cryoglobulinemia and hypocomplementemia but no ANCA were present. Renal biopsy revealed MPGN and he was partially remitted under antimicrobial and corticosteroid therapy [27]. In such circumstances, renal biopsy was obligatory to clarify the diagnosis, guide the treatment and predict the prognosis. The diversity of pathological change shows the complexity of renal immunity for the individual. Also, we could learn from these cases that acute glomerulonephritis could happen in any phase of brucellosis, even in a clinically silent course of chronic brucellosis infection.

Antimicrobial treatment is the cardinal treatment for glomerulonephritis associated with brucellosis. In all the reports in the literature, specific antimicrobial agents mainly doxycycline, rifampin, and sometimes the trimethoprim/sulfamethoxazole cefotaxime were given in a longer time range from 3 months to 12 months for most glomerulonephritis occur in brucellosis infection [5, 19, 28]. As an intracellular bacterium, the eradication of Brucella needs long-course antibiotic treatment ranging from 6 weeks to a year and the chronicity of brucellosis is the consequence of the inadequate treatment of the first attack [29, 30]. In our case, 6 weeks regime with doxycycline and rifampin was applied each time with persisting existence of a positive antibody which was thought to be inadequate long enough. The renal pathology revealed that the frequent relapse of brucellosis infection may the foremost reason of the renal injury in our case. The reason for specific antibiotic treatment lies in the following two concerns: For one thing, the persistent positive SAT may indicate a chronic infection of *Brucella*, even when there were no signs of active infection. For the other, the administration of corticosteroids may elicit the recurrence of *Brucella* infection. Therefore the specific antimicrobial treatment for brucellosis is the cardinal treatment for the complications of infection even if the patient has no sign of active infection and longer courses are needed in chronic cases, especially when corticosteroids were given.

In most cases of brucellosis associated with renal injury, specific antimicrobial agents for Brucella bacteria are sufficient to make remission, which includes IgA nephropathy, and diffused mesangial proliferative glomerulonephritis or acute renal failure cases [5, 31–33]. But some of the prognosis of renal involvement associated with brucellosis was rather poor without corticosteroids [34]. The application of corticosteroids improved the prognosis of renal injury associated with brucellosis. The corticosteroid was applied when the specific antimicrobial agent didn’t work or when the patient was accompanied by rapid progressive renal insufficiency, nephrotic proteinuria, aortitis or accompanied with vasculitis [27, 35–37]. The patient usually had a good response to corticosteroids. The dose of corticosteroids varies from 0.5 mg per kg body weight to methylprednisone 500 mg for impact therapy. In our case, prednisone 50 mg/day was given for 4 weeks with a good response to the treatment. The proteinuria decreased dramatically and the diuretic effect appeared and renal function recovered. Then prednisone was tapered with 5 mg per week. The mechanism of corticosteroids is due to the immune abnormality participating in the pathophysiology of renal injury associated with brucellosis [4, 38].

The coexistence of ANCA and cryoglobulinemia was not common. Both cryoglobulinemia and ANCA may be induced by infections [39, 40] and both may cause vasculitis [41, 42]. While the mechanism, clinical features and the pathological manifestations of the two circumstances are quite different [43, 44]. So, it is unusual for the two kinds of immunological abnormalities to coexist in one patient. But the coexistence of them does present in clinics, especially in chronic infections [45] such as HCV infection [46] or subacute bacterial endocarditis [9]. In 1998, Lamprecht et al. reported two cases of vasculitis-associated glomerulonephritis patients in whom both c-ANCA and type II cryoglobulinaemia were detected. One of the cases was associated with hepatitis C virus (HCV) infection. The patient developed nephrotic syndrome and AKI besides purpura, and had a good response to interferon-α treatment, but showed no effect to the initial prednisone treatment [44]. Bele et al. reported an unrecognized subacute bacterial endocarditis associated with ANCA-positive immunocomplex glomerulonephritis, accompanied by cryoglobulinemia. The patient completely recovered after the combined use of glucocorticoids, antibiotic therapy and surgical valve repair [9]. A recent study revealed Bartonella infection could also induce immune-related glomerulonephritis with the coexistence of ANCA and cryoglobulinemia and subacute endocarditis [47]. Although there are some similarities in clinical features, the pathological changes and prognosis in different infection-related vasculitis varied from one case to another. So it should be treated case by case. It is thought that the concomitant presence of ANCA and cryoglobulins in prolonged infection patients was associated with severe glomerulonephritis in ANCA- associated infection patients [45]. Other cases of the coexistence of ANCA and cryoglobinemia were reported in blood malignancy [48], autoimmune diseases [49] and medicine induced [50]. In our case, the coexistence of ANCA and cryoglobulinemia were present in a chronic brucellosis patient, which is not ever reported in English literature. The mechanism of the coexistence of cryoglobulinemia and ANCA induced by chronic infection is not fully clarified. But it has been suspected to be related to the activation of polyclonal B lymphocytes, which in turn produce multiple autoantibodies, including ANCA, cold globulin and vascular damage which may stimulate the expression of the cytoplasmic enzyme in endothelial cells and polymorphonuclear giant cells under the stimulation of prolonged infection [44].

Aortic involvement is a rare complication of brucellosis infection, which is due to hematogenous spread or is contiguous from either adjacent infective endocarditis or spondylitis [51, 52]. Brucella aortitis and aneurysm formation are the common manifestation and combined prolonged triple antibiotic therapy and surgical resection are treatment principles. In our case, the left iliac aneurysm was diagnosed in 2014 when he worked as a shepherd. But the brucellosis was not diagnosed then and the aneurysm was thought to be the result of hypertension and resection and stent implantation surgery were performed. But we still suspected brucellosis infection was the cause of the abdominal aneurysm because brucella aortic aneurysm may result from hematogenous planting of atheromatous plaques [36].

What can we learn from our case? The coexistence of ANCA and cryoglobulinemia should arouse the suspect of infection-related glomerulonephritis even when there is no obvious sign of acute infection, rather than just taking it as AAV. Brucellosis is a rare cause of it. Past medical records are important in making a proper diagnosis. Renal biopsy is critical for the diagnosis of APIGN or idiopathic AAV. The specific antibiotic treatment is the fundamental treatment for APIGN that coexist with ANCA and/or cryoglobulinemia. If it doesn’t work, combined therapy of corticosteroid with antibiotics should be applied to improve the prognosis of the renal injury. The duration of antibiotics should be long enough to avoid relapse.

## Electronic supplementary material

Below is the link to the electronic supplementary material.

Supplementary Material 1

Supplementary Material 2

## Data Availability

The datasets used and/or analysed during the current study available from the corresponding author on reasonable request.

## Abbreviations

AAV: ANCA-associated vasculitis
ANCA: antineutrophil cytoplasmic antibody
APIGN: acute post-infectious glomerulonephritis
CRRT: continuous renal replacement therapy
CT: computed tomography
HCV: Hepatitis C virus
MPGN: membranoproliferative glomerulonephritis
SAT: serum agglutinins test
WBC: white blood cells.
