# Supplementary material for: Coexistence of cryoglobulinemia and ANCA-associated vasculitis in a chronic brucellosis patient -a case report and literature review
Source: BMC Infect Dis. 2023 May 2;23:272. doi: 10.1186/s12879-023-08232-w (PMC10152744; doi:10.1186/s12879-023-08232-w)
Supplement: Supplementary file 1 — Supplementary Material 1 [file 12879_2023_8232_MOESM1_ESM.docx]

Supplemental material.


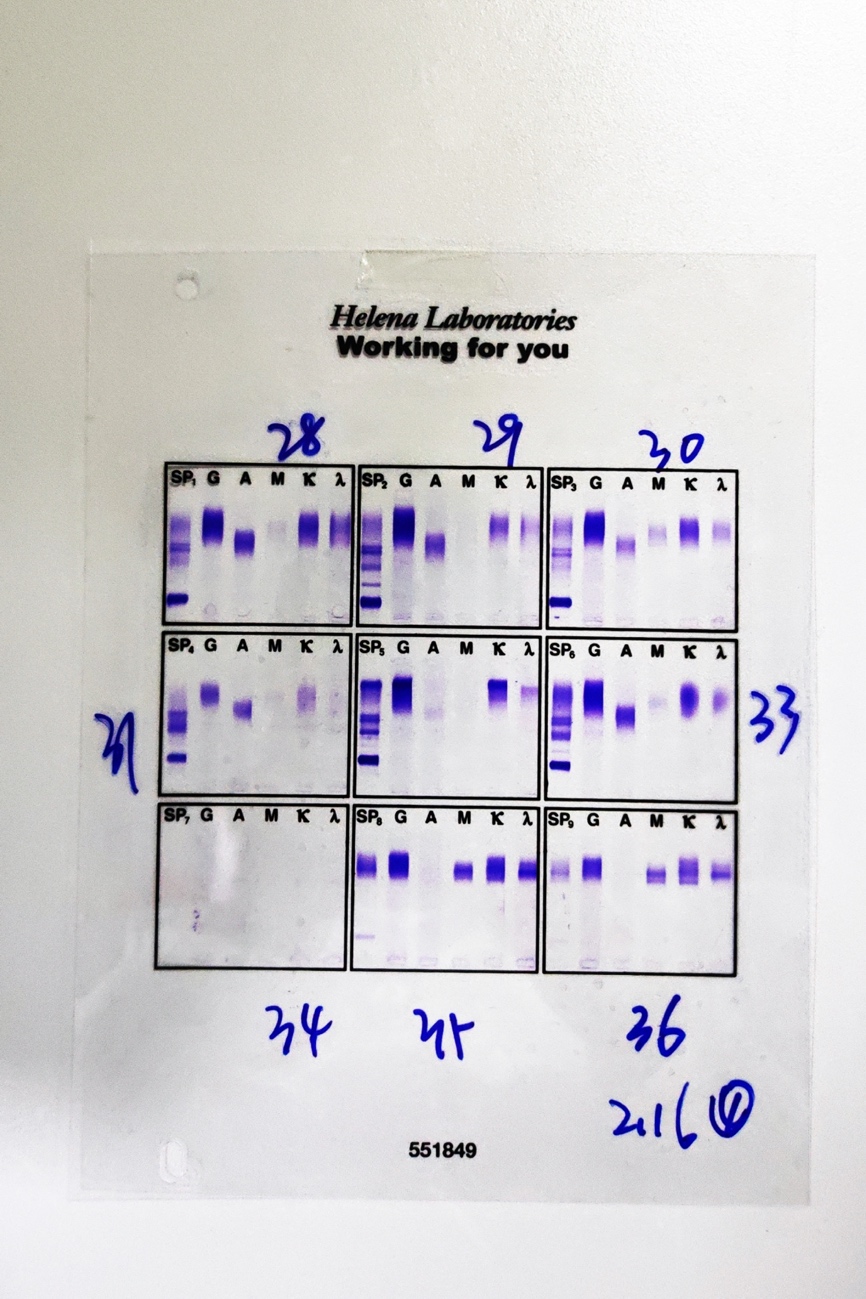


Fig S1. The original membrane of Fig 2C and D. In this membrane, our cryoglobulin samples of the patient labelled SP8 & SP9 were loaded simultaneously with other patients’ samples.

We upload a 300dpi original Fig 2C and D in the supplementary material.

We used a commercial method by Helena Laboratories (USA) to perform the immuno-electrophoresis currently used in our clinical laboratory centre. Immuno-electrophoresis of the cryoglobulin was performed with a monoclonal immunoglobulin kit (electrophoresis-immunofixation method) by Helena Laboratories (USA), which includes 5 kinds of antiserum to monoclonal immunoglobulins (IgG、IgM、IgA、Kappa、Lamda). All the procedures were performed according to the instruction (<https://www.helena.com/Procedures/Pro200Rev7.pdf>).

Duplicates were not performed for we thought the difference between that before and after treatment was apparent not only grossly, but also supported by the quantitative measure and the purpose of the immuno-electrophoresis was to identify the components of the cryoglobulin.
