# Supplementary figures and images for: Coexistence of cryoglobulinemia and ANCA-associated vasculitis in a chronic brucellosis patient -a case report and literature review
Source: BMC Infect Dis. 2023 May 2;23:272. doi: 10.1186/s12879-023-08232-w (PMC10152744; doi:10.1186/s12879-023-08232-w)

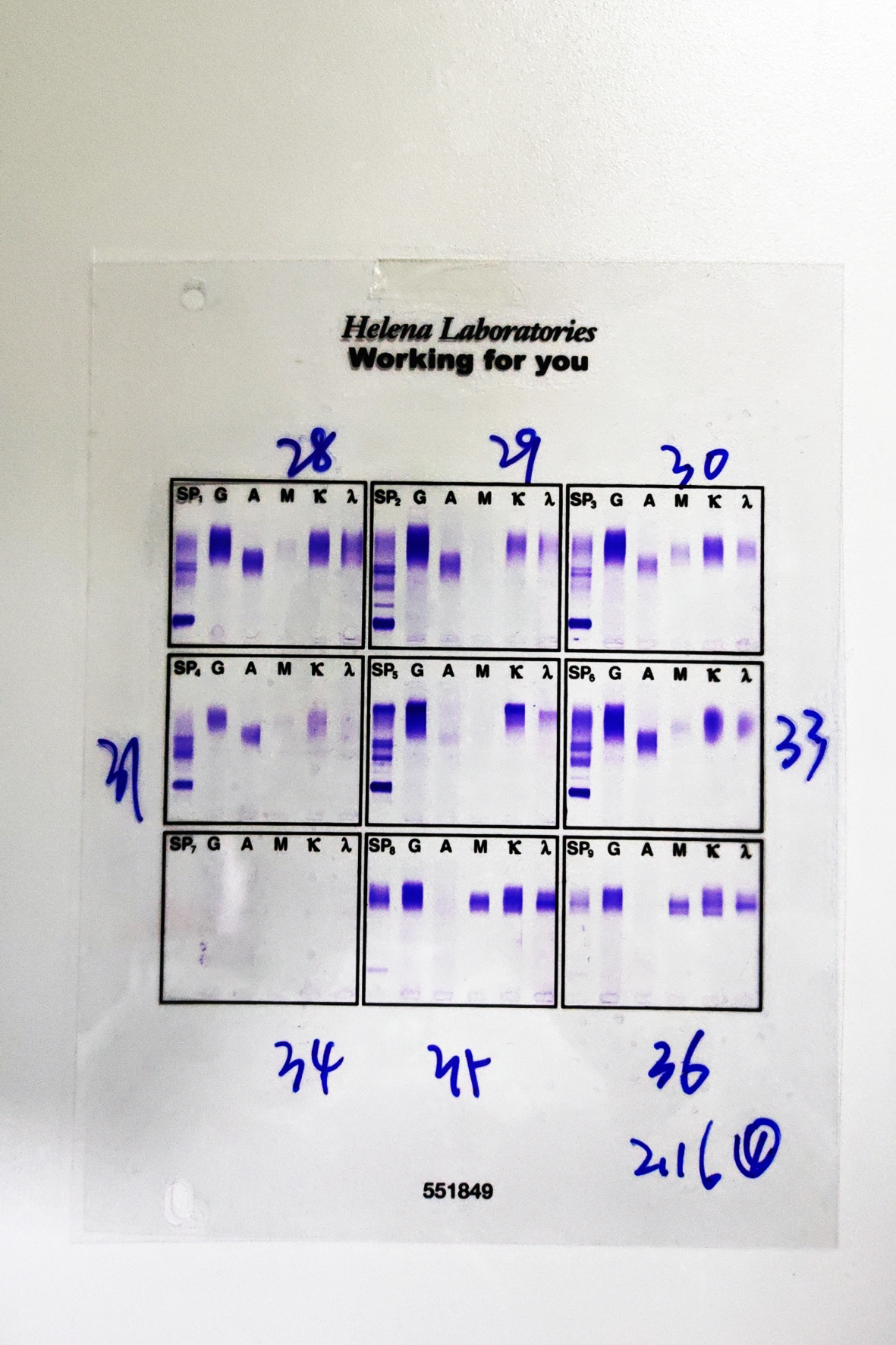

Supplement: Supplementary file 2 — Supplementary Material 2 [file 12879_2023_8232_MOESM2_ESM.tif]
